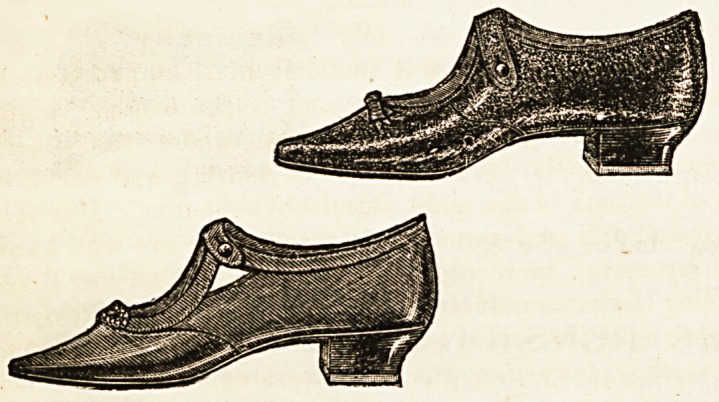# The Hospital Nursing Supplement

**Published:** 1895-07-27

**Authors:** 


"The Hospital" Nursing Mirror.

Being the Extra Nursing Supplement of "The Hospital" Newspaper.

[Contributions for this Supplement should be addressed to the Editor, The Hospital, 428, Strand, London, W.C., and should have the word "Nursing" plainly written in left-hand top corner of the envelope.]

### News from the Nursing World.

### "OUR HOSPITAL."

"Whatever's up there?" asked a stranger from the country, who was seated on the top of a tram, apparently the only part of the vehicular traffic unsuspended in Gray's Inn Road on Monday last. "Well, if yer wants to know," retorted his neighbour, "the Prince of Wales an' the Princess is a-drinkin' tea in *our* hospital. I seen 'um go in with their daughters; and," he added, condescendingly, "if you manage to look a bit sharp, young man, you'll see 'um come out." The eager countryman took the hint, and, swiftly alighting, he became another unit in the patient, orderly crowd of workers who lined both sides of the long roadway. "Our hospital," as the people call it, stands in the centre of a vast population to whom "our nurses" are familiar figures, claimed as friends by a succession of patients to whom the great doors of the Royal Free Hospital are ever open. Excellent and varied is the experience gained in the long, bright wards; and it is to be hoped that the next addition to the building at the Royal Free Hospital will provide a nurses' home as complete as the new structure of which the formal opening is fully reported this week in our columns.

### AT THE GREAT NORTHERN.

The recent endowment of a bed at the Great Northern Central Hospital is entirely due to the energy of the Ladies' Association. By their exertions a sum of £1,050 has been raised to create a perpetual memorial of Dr. William Cholmeley, one of the original founders of the hospital, who for forty years has devoted himself to the interests of the institution. The inauguration of the bed was made the occasion of a pleasant meeting at the hospital last week, the chair being taken by the Hon. Reginald Capel, who expressed the feeling of all present when he commended the erection of so enduring a memorial in Dr. Cholmeley's own lifetime.

### GRATEFUL ACKNOWLEDGMENTS.

"Nurse is looking very much better and stronger, and they were most kind to her at the home. The doctor is quite pleased, and thinks she ought to go on well now, if she takes reasonable care. It therefore seems unnecessary for her to have a longer rest. Some other delicate nurse is certain to be anxiously waiting to enjoy advantages similar to those granted by The Hospital Convalescent Fund in her case. Rest, kindness, and fresh air without expense are benefits which perhaps only a nurse with limited means and health can estimate at their full value." This is the substance of the letter of thanks written by the matron of a cottage hospital, and she concludes: "I thank you in nurse's name, and in my own, for her holiday. I do not know how we should have managed without this help." Such a case as this is one of the most satisfactory which the Fund can take up. It means the restoration to health of a nurse who, before this restful month at the sea, was absolutely unfit for duty, and with no means to provide the much-needed holiday. Is not the bestowal of assistance such as this worthy of our readers' support? By it valuable workers are placed in the best condition for carrying on labours which benefit alike the rich and the poor in their sicknesses. We shall be glad if those wishing to "help those who help themselves" will forward their subscriptions to the hon. secretaries of The Hospital Convalescent Fund, 428, Strand, London.

### THE FIRST AND SECOND THOUSAND.

"But what about us?" ask the First Thousand, "cannot we also wear the badge approved by 'Our Princess'? We are so anxious to know, and the Second Thousand are just as curious as we are about it." If the First and Second Thousand will have a little patience (they will not need much), they will have good news about the armlet which "Our Princess" has sanctioned all policy-holders wearing as long as they belong to the Royal National Pension Fund. It is not possible for the whole number to be got ready this week, and therefore it is only reasonable that the first to receive them should be the recruits invited to Marlborough House, but the rest will be issued and forwarded to the First and Second Thousand without a moments avoidable delay.

### A NEW LADIES' LEAGUE.

A Ladies' League has been established in connection with Guy's Hospital, having for its primary objects the provision of clothing for destitute patients when they are discharged, and the visiting of in-patients "at times convenient to the authorities." At the first meeting, which was held on June 28th, it was decided that rules and regulations should be drafted by a committee appointed for the purpose. The next meeting will take place in October.

### HAMPSTEAD DISTRICT NURSING.

The drawing-room meeting recently held at Mr. Fletcher's at Hampstead was well attended, and the cause of the local district nursing association was ably advocated by Miss Warwick, a Queen's Nurse, and by the Rev. P. L. Peile, President of Queen Victoria's Jubilee Institute. The Hampstead association, founded ten years ago, was affiliated with the Institute in 1890, and now employs three duly trained nurses. There appears to be ample work for more nurses, and the wealthy inhabitants of Hampstead will doubtless secure, by increased subscriptions, a larger staff for the sick poor in their own homes. The financial position of the association at present is by no means satisfactory, for, according to the report in the local press, the expenditure exceeds the contributions.

### WOMEN DOCTORS.

The Council of the Royal College of Surgeons has received a petition from the London School of Medicine for Women, asking that women be admitted to the examination for the diploma of membership. It is probable that the Committee of Management, to which the petition has been referred, may advise compliance with this request.

### FANCY FEES.

"I don't think it's commonly honest," remarked a private nurse. She had been telling a friend that the institution to which she belonged had charged "infectious fees" for every case of real or supposed influenza to which a nurse had been sent. Yet the latter never underwent any period of quarantine before going to another case, nor did she derive benefit from the exorbitant sums charged for her services, being paid a fixed and moderate salary. "Of course the patients and their friends grumbled, but what could they do?" concluded the nurse. It seems to us that the remedy for such things lies in the employers' own hands. They should acquaint their doctors with facts which they would be the first to condemn. Exposure of such practices would soon, we believe, put a stop to them.

### SOCIETY OF TRAINED MASSEUSES.

A friendly little gathering took place last week at the Trained Nurses' Club, 12, Buckingham Street, on the occasion of the distribution of certificates to pupils who had passed the examination held by the Society of Trained Masseuses. These certificates, very well got up on vellum, are only secured by candidates who succeed in thoroughly satisfying three examiners, and they appear to be valued in due proportion to the difficulties of attainment. After the distribution, which was followed by one or two short speeches, the candidates and their friends were entertained at tea with the hospitality which has always characterised the Council of the pleasant Buckingham Street Club.

### COMBINED DAY AND NIGHT DUTY.

No week passes without attention being called to some place or other where an overworked day nurse is called up at night. It sounds a small thing, but it is really a very serious matter to the nurse who has to work month after month, and year after year. "She isn't often disturbed at night, I believe," remarks a complacent member of committee, who would think herself vastly ill-used if her long night's rest were interrupted even once in a month. In cottage hospitals, and in workhouses with only one female nurse, the latter can never count on an unbroken night. It must be borne in mind that the day most full of anxiety and work for the nurse is the one to be followed by the watchful and wakeful night. To lose one night's sleep is a small matter, but to be condemned every night to attend to each summons which may come, is a trial only measurable by those who have undergone it. Any employer who makes light of the strain, when borne by other people, is advised to make a personal trial of one month of broken nights, combined with long working-days!

### NURSES FOR COUNTRY VILLAGES.

This prefix of "country" is apparently used, by a writer in *The Parish Councillor*, to emphasize the rural aspect of the villages for which this article claims the reader's pity. In seeming ignorance of the presence to-day of trained district nurses in many English, Scotch, Irish, and Welsh counties, this correspondent naturally approaches the matter at grave disadvantage. The remedy for all ills, such as lack of sanitation and women's wasted lives, will be found, it appears, in the nurse of three months' experience. Now, granting that a villager may be the best material for a village nurse, she certainly requires at least as much instruction as her quicker-witted town sister. Therefore, instead of creating this cheap substitute for a nurse, the philanthropist of the country should advocate adequate training for villagers, to make them if possible the equals of the "Health Missioners" and the district nurses, already well-known and respected members of many country communities.

### MEDALS FOR FRENCH ATTENDANTS.

The fine buildings of the Hotel Dieu, so familiar to every visitor to Paris, were recently inspected by the President of the French Republic. His presence in the hospital gave much satisfaction to the staff, and was made memorable to the male and female attendants by eight of their number receiving medals at his hands.

### PINAFORES AND POLITICS.

"Which way are you going to vote?" asked a white-faced boy, anxiously. The visitor laughed at the unexpected question, and looked again at the small occupant of a bed in a Home for Sick Children. "I've done my voting already, Tommie," he answered; "but what do you know about elections in here?" Several eager little faces looked quickly up at the questioner. "Of course we know," they said in chorus; "we're every one of us Conservatives, too." "How's that?" asked the amused visitor. "Well," said Tommie, "you see our chairman's the Conservative candidate, and as he always gives us better Christmas boxes than anybody else, we're going to stick to him!"

### THE YARROW HOME.

The invitations which have been issued for the formal opening of the Yarrow Home at Broadstairs are accompanied by an intimation that donations and subscriptions are neither invited nor will be accepted. This beautiful home is designed to accommodate 100 children, whose ages the trustees have fixed between four and twelve years. Five shillings weekly will be charged for each, and they are to be selected from the middle-class rather than from the very poor. The children of the clergy, clerks, and others with limited incomes will be eligible so long as they are not chronic cases. The Yarrow Home is intended for delicate children, to whom a three weeks' seaside visit means complete restoration of health.

### QUEEN'S NURSES IN SCOTLAND.

The nurses who have been recently added to the roll of the Scotch Branch of the Queen's Jubilee Institute will receive their badges in the autumn. It is possible that they will be distributed by Her Royal Highness Princess Louise, president of the Scotch Branch, whose interest in nurses and practical knowledge of nursing are alike known and appreciated by our readers.

### THE ROYAL BRITISH NURSES' ASSOCIATION.

The annual meeting of the Royal British Nurses' Association has taken place, Her Royal Highness Princess Christian being received on arrival with much enthusiasm. A report of the meeting, luncheon, and the afternoon reception held by Her Royal Highness will be reported next week.

### SHORT ITEMS.

The work of the Queen's Nurse at Enniscorthy has been carried on more successfully under the auspices of the District Nursing Association.

### Elementary Anatomy and Surgery for Nurses.

By W. McAdam Eccles, M.B., M.S., F.R.C.S., Lecturer to Nurses, West London Hospital, &c.

### XXVI.—THE SPECIAL SENSES.

(*Concluded from page* cv.)

The sense of *Hearing* has for its special organ the Ear. The ear consists of three parts: The external ear, the middle ear or tympanum, and the internal ear or labyrinth. The two former merely conduct sound, the last receives the impressions upon the endings of the auditory nerve. The outer ear consists of the pinna, which projects from the side

Fig. 39.—Section of the Ear.

of the ear, but is nearly functionless in man, and the external auditory canal blocked at its inner end by the tympanic membrane or drum of the ear. This canal runs inwards and slightly forwards, but is not quite horizontal, there being an elevation in the floor near the middle. (See Fig. 39.) Hairs grow from the wall of the canal, and wax is secretion from the glands contained in the tissues forming the wall. The middle ear is a small cavity in the temporal bone separated externally from the auditory canal by the tympanic membrane which is very obliquely placed, but communicating by a

Fig. 40.—Section of Circumballate Papilla showing Taste Buds.

narrow passage, the Eustachian tube, with the pharynx. Through this latter air passes into the tympanum. Three very small bones, the auditory ossicles, are found within the middle ear. These articulate with each other, the outer being attached to the inner surface of the tympanic membrane, while the innermost fits into an opening in the wall between the middle and internal ear. (See Fig. 39.) The internal ear is too complex to warrant a description here. Suffice it to say that it consists of channels filled with fluid, and has the terminations of the auditory nerve distributed upon its membranous walls.

The sense of *Taste* has the tongue as its chief organ, and this has already been briefly dealt with in Lecture XII. It may be added that the endings of the glosso-pharyngeal nerve can be traced to peculiar bodies called taste buds, which are found in the walls of the moat around the circumvallate papillæ at the back of the tongue. (See Fig. 40.)

Fig. 41.—Vertical Section of Skin Showing Glands.

The sense of *Smell* has the nose as its special organ, which has been alluded to in Lecture III. It should here be remarked that it is only the upper part of the nasal cavities which are concerned with this special sense, for it is to this part that the terminations of the olfactory nerves pass.

The sense of *Touch* resides for the most part in the skin. In addition to the skin being the seat of the nerve-endings forming the essential part of the sense of touch, it also has glands which are of two kinds, the sebaceous and the sweat glands. Growing from it, moreover, are the hairs and nails. A good idea of the structure of the skin may be gathered from reference to the accompanying diagrams. (See Fig. 41.)

### National Health Society.

In October, the National Health Society recommences its special courses of training lectures for ladies who wish to qualify as teachers of hygiene and nursing, under the County Council scheme of technical education, as factory or sanitary inspectors, as lady lecturers, or in preparation for hospital training. By removing the age qualification from the syllabus of the National Health Society's training course, the course has been thrown open to ladies too young to enter a hospital, thus enabling them to go through preliminary training. In addition to this the lecturers' age has been lowered from 25 to 23 years, and this enables ladies not only to qualify but to occupy two years of the waiting time by profitable lecturing. All further information can be obtained by application to the Secretary, 53, Berners Street, Oxford Street, London, W.

### Nursing in Japan.

### UNIVERSITY HOSPITAL, TOKIO.

By a Private Patient.

This hospital is attached to the Imperial University of Japan, and the head surgeon and physician being Germans, everything is carried on in the German style. The hospital consists of a number of bungalows connected by closed verandahs, and I believe there are about 300 beds.

Being a private patient I have a room about eleven feet square, and on first taking possession of it the furniture consisted of an iron bedstead, one chair, a three-cornered washstand, a little bed-table, whilst a little looking-glass hung upon a nail. Since then I have made myself more comfortable by getting crockery, knives, forks, spoons, towels, glasses, an easy chair, &c., &c., from Yokohama. The table I write at was left behind by the last foreign patient, who had it made for him.

All the nurses are females, and a funny lot of little creatures they are. I have a probationer to myself at an extra expense of 18 cents (about 4d.) a day. She is not quite four feet high, but quite two feet wide, and the personification of good humour. The uniform consists of a kind of ulster made of white cotton cloth, buttoned up to the throat and reaching down to the ground and tied round in the middle. Being worn over the ordinary native dress, the nurses look like so many bolsters or animated clothes-bags. Seven of them take it in turn to look after me, and they all, I think, belong to the peasant class except one, a ladylike, graceful, but plain girl, who is more reserved than the others, whilst her dress looks clean even towards the end of the week. They are all very careful and attentive, and seem to take great interest in the cases; but it is like playing at nursing, and I feel as if I were in a dolls' house. If I ask about any other patients, who may be dying, perhaps, they try to look serious for a moment, but soon burst out laughing, which would be trying to a very sick person; but the Japanese, as a rule, are not sensitive.

A little before five a.m. I am wakened with the noises familiar to those who have lived in Japanese tea-houses. Doors and shutters are banged, the day nurses arrive and scuttle about, laughing and joking—so there is no more chance of sleep. At quarter-past five a nurse comes in to take my pulse and temperature. At half-past six a man walks through the passages banging two pieces of wood together; this is the announcement of breakfast. All the nurses rush away to a large room, where each person's food is served out by messengers from a neighbouring tea-house. It consists of rice, a very unpalatable fish called tai, some salted plums, pickled radishes, a little soy, and tea. Patients upon low diet have soup and milk, which are the only things cooked on the premises.

After this uproar is over we have comparative quiet for an hour or so. Then the visiting surgeons commence their rounds and the operations come off. My window looks across a courtyard into one of the passages, and I can see the patients going to the theatre, carried on a kind of truck. The theatre is a fine large room, with raised seats for at least 50 or 100 students. I had never been in one before, but what struck me as very curious was a full-sized skeleton within six feet of the operating table, so placed that it is the last thing the patient is sensible of. There is certainly no lack of attention. Besides the operating surgeon, there is a Japanese on each side feeling the pulse and another behind administering the chloroform, besides a dozen nurses and other attendants.

About eleven o'clock medicine is served out by a man who keeps on yelling at the top of his voice for an hour or more. I suppose he calls the names of the nurses. At twelve comes dinner, which is the same as breakfast, and I am now allowed to go out for a little walk about four o'clock.

The grounds are very pretty, having formerly been the residence of a native prince. There is a pond with rocks, an island, and some fine old chestnuts, but everything is neglected, except just where the ground is cleared away for the university buildings. These are numerous, some being substantially built of brick and stone. At five p.m. there is supper, same as dinner. Between nine and ten all the day nurses go away, after much laughing and scuttling about. They must be having a game of leap-frog, except that their costume would interfere with them. Then the hospital is left comparatively quiet for the night. In addition to the ordinary noises there are visitors coming and going all day, and some carpenters are at work a few feet from my room, who keep up an incessant hammering from early morn till dewy eve. They do not seem to have made much progress in the last three weeks, and must, I think, be employed to keep the patients lively! But, as I said before, the Japanese are not sensitive to sound.

All the surgical appliances are very good, everything being beautifully clean (except, sometimes, the nurses' dresses). My doctor told me they have all the latest improvements, and that, except for the little drawbacks above mentioned, I am as well off as if I were in a London hospital.

I can eat anything I like, and as I do not fancy Japanese food my mess is a daily interest. A tea-house in the neighbourhood supplies me with very good, though monotonous, soup, bread, and eggs, and from another eating-shop I get stewed eels; anything beyond this has, so far, proved a failure. A friend has, however, sent me some chicken pies, and my friend in Yokohama has supplied a ham and some fowls, so I am doing very well. All the food has to be kept in my bed-room, so it is like being on a picnic. Strawberries have just come into season, and I can eat any quantity of them, and tea and coffee are sent me from Yokohama. I am the only foreigner here now. A friend of mine from Nagasaki came in a few days after me, and was operated upon for cancer of the tongue, but he died the day after the operation.

The nurses are laughing at a Japanese lady in the next room to mine, who is able to walk about. This morning she thought she would like to go into the theatre and see some operations. The result was that she had to be carried back to her room, where they are now putting ice on her head. If you would like a few nurses from here you can easily get them. They are all ready to go.

### Notes and Queries.

### Queries.

(210) *Badges.*—Are not the first and second thousand going to have the armlets as well as the third and fourth thousand? The Princess's colours are equally desired by us all.—*A Policyholder.*

(211) *R. N. P. F.*—Can you tell me why the delightful evening party which preceded the two former Marlborough House garden parties has been discontinued? The last one was the most enjoyable evening I ever spent in my life.—*Discontent.*

(212) *Crutches.*—I shall be very glad of advice about the young man whose case I have fully explained in my letter.—*R. H. W.*

(213) *Institute.*—Your kindness in replying to so many nurses' questions every week encourages me to ask for advice. I am thinking of opening an institution for private nurses in a London suburb, if you will tell me where one is wanted. I am a trained nurse, and have had experience in temporarily managing such a place for a friend.—*M. J.*

(214) *Abroad.*—Can you advise me of nursing institutes in the South of France or North Africa. The Holland Institute will not require any fresh nurses till December, and I want to go out before that.—*H. L.*

### Answers.

(210) *Badges* (*Policyholder*).—Certainly, the armlets will be the badge of *all* the Royal Pension Fund Nurses. Read first page of this supplement.

(211) *R. N. P. F.* (*Discontent*).—It is thought more convenient for the preliminary meeting to take place on the same day as the reception at Marlborough House. We quite agree with you as to the pleasure of those two delightful evenings when the founder of the fund entertained us so magnificently.

212. *Crutches* (*R. H. W.*)—We should think that the National Hospital, Queen's Square, London, would take the case. Write full particulars to Mr. Burford Rawlings, the Secretary, and ask for rules regarding admission, &c.

213. *Institute* (*M. J.*)—Without plenty of capital you should not dream of such an undertaking. Constantly do nurses risk their hard-earned savings in this way, and generally end in failure and distress. A nurses' home might be made self-supporting if there were enough members to make it so. A house of moderate dimensions in a suburb would not be a profitable venture for you if the nurses received their own earnings, as they ought to do. If you study the tariff of such homes you will see what very moderate prices are named.

114. *Abroad* (*H. D.*)—You will find other institutions mentioned in Burdett's "Hospital Annual, 1894." When there are vacancies in any of the hospitals on the north coast of Africa, they are generally advertised.

### The Royal National Pension Fund for Nurses.

THE CERTIFICATE OF THE THIRD AND FOURTH THOUSAND NURSES.

By Arthur Hacker, A.R.A.

### THE NEW CERTIFICATE.

We think all nurses will like to see the certificate which H.R.H. the Princess of Wales is presenting to the third and fourth thousand members of the R.N.P.F.N. on Friday. We believe all will agree that the new certificate has been charmingly designed by Mr. Arthur Hacker, A.R.A., who presents the beautiful drawing to the fund as a token of interest in its objects. Mr. Hacker has most appropriately chosen Sympathy for his subject. Sympathy is represented with ample sheltering wings, the graceful form expressing repose and humility, though glorified by a halo round her head. The lines of the drawing are simple, but fully expressive of those noble attributes which should always be the handmaid of nursing, and without which the greatest skill loses its value. The rest of the certificate is well carried out, "Our Princess's" monogram forming an effective finish, and the whole is printed in a deep tone of red. The certificate cannot fail to be a valued possession, especially to those who will connect it with the memory of their gracious President's kindness. As an artistic production it will prove a pleasing addition to the adornments of our nurses' rooms.

### Friend, not Busybody.

The pleasant social gathering recently held in connection with the Southwell District Nursing Society was attended by members of all religious denominations. The proceedings were commenced with a tea party in the National Schools, after which an adjournment was made to the Concert Hall. The work of Nurse Stiffe, who was supplied by the Queen's Jubilee Institute eight months ago, has been uniformly satisfactory. One of the speakers is reported to have remarked: "Some had the idea that a nurse was a gossiping busybody; they had found that was not the case, but that she was a sincere friend, who efficiently attended to her duties."

### The Registration of Midwives.

At the forthcoming meeting of the British Medical Association in London, Mr. Lawson Tait and Mr. Colin Campbell will bring forward a motion against the Midwives Registration Bill. The honorary secretary of the Midwives Registration Association is desirous that all interested in registration should attend a meeting to vote and speak against this motion of Mr. Lawson Tait, in Exeter Hall, on Tuesday, July 30th, at half-past two. Doubtless a large number of persons who appreciate the importance of this subject will make a point of being present.

### Novelties for Nurses.

### NOVELTIES IN BOOTS AND SHOES.

No woman can be really well dressed who is neglectful of the appearance of her feet. This important item has been recognised by the London Shoe Company, whose catalogue affords a most tempting display of dainty articles. A visit, however, to their establishment (116-117, Bond Street) is what we should recommend to those who can manage it, as the choice is larger and more varied even than that contained in the pages of the price list. In appearance and cut these popular goods are equal to those turned out by the best firms and at about one-half the cost. It is more especially towards those designed for nurses that we would turn our attention, two shapes in particular claiming admiration. The stouter of these samples is made in morocco leather, with a strap that crosses over the instep and a square military heel capped with indiarubber to deaden sound. They are cut low in front and are very soft and pliable, thus fulfilling the most fervent aspirations for something comfortable as well as becoming. The lighter pair is in very fine glacé kid beautifully finished off, and ornamented in front with cut steel buttons. They are smarter-looking altogether than the first pair we have described, and though lighter, will no doubt prove equally durable. One great advantage of all articles made by this firm consists in their being kept in half sizes, which ensures a perfect fit being obtained. Those of our readers who are wanting nice-looking shoes for walking as well as ward wear, cannot do better than go at once to be fitted.

### A NEW CAP.

Messrs. J. R. Roberts' Stores (Broadway, Stratford, E.) are at present showing an extremely becoming and convenient nurse's cap. It is made of nainsook, triangular in shape when flat, and edged in front with a gophered fancy frilling, which graduates in width from the centre to the sides. A runner goes all round the back about a couple of inches from the edge, which is turned up with a narrow hem, and a tape draws the cap into the required position after its return from the laundry. Narrow strings edged with gophered frilling round the bottom are fastened at either side, and can either be tied in a bow under the chin or hang loose down the back, according to the taste of the wearer. We have no hesitation in recommending this cap to the attention of our readers, as from its neatness, convenience, and cheapness it only requires to be known to obtain an extensive and well-merited patronage.

### A PERFECT DOUCHE.

Messrs. Hockin and Wilson are now supplying the most complete douche we have yet seen. The form is the same as is usual to douches, made to hang on the wall. The one before us is made to hold four pints of liquid. Let into grooves running down the front are thermometer and measure. The measure is a simple contrivance consisting of a glass tube containing a ball, which rises and indicates on a scale at the side the amount of liquid which is entering the receptacle. The advantage of this ready means of ascertaining both quantity and temperature needs no pointing out. The douche will be most popular owing to its convenience and moderate price. Messrs. Hockin, Wilson and Co.'s address is New Inn Yard, 186A, Tottenham Court Road.

### PURE WOOL UNDERCLOTHING.

At first sight it would appear hardly necessary to draw the attention of our readers to warm underclothing at this season of the year. Practical experience, however, teaches us that at no time is it so dangerous to adopt light underclothing as at the commencement of an English summer. Manufacturers are now alive to the necessity of providing all thicknesses of woollen materials, and so no difficulty exists in modifying without entirely discarding our woollen undergarments. Whilst admitting the advantages of wool from a sanitary point of view, some persons raise objection that æsthetically such underclothing is a failure. This reproach cannot be applied to the articles manufactured by Messrs. Roberts Harrison, of Castle Donington, Derby. The firm has succeeded in producing quite the most dainty and tasteful woollen underclothing we have seen. Made of the most beautifully soft and elastic material, the prettiest white and pink smocked garments are to be bought at very moderate prices. A price list (with specimens of various qualities of materials in which the garments are made) is well worth inspection, and so various are the items on Messrs. Roberts Harrison's list that it is impossible to enumerate them here. All classes and all ages of the community will find their wants considered, and attractive or plain woollen underclothing is brought within the reach of most purses. Now is a good time to buy, as, owing to a reduction in the wool market, Messrs. Roberts Harrison are allowing a discount of 5 per cent. off their goods.

### DRESS NOVELTIES.

Serges and homespuns are more in demand than ever, indeed, their popularity seems but to increase as time goes on. Mr. Egerton Burnett is, as usual, to be found in the van of fashion, and deserves our warmest congratulations on the charming and comprehensive nature of the patterns he is now sending out of spring novelties. Serge in every variety and shade is offered at a most reasonable price per yard, and the famous "Royal" serge among them in both black and blue in several different qualities. To see is to admire, and to admire is the first step towards purchase. Once having decided on the material, all difficulties with regard to the making up are obviated by the convenient establishment of a high-class tailoring department, where any style of dress can be made from a simple form of self-measurement, for which full instructions are given. The beauty of serge is that it never goes out of fashion, and is one of the few materials that does not "carry date." If properly cut the serge gown of to-day is as smart as that of twenty years ago, when, as a material, it first became the rage. We would call special attention to a fine close make which can be had in either black or navy, and is admirably adapted for summer costumes or cloaks. There is also a good selection of waterproofs, a small black and white shepherd's plaid being especially *chic*. We are glad to observe that washing materials for nurses' uniforms are included in this excellent assortment. Some of these are charming in the extreme, and few will be able to resist a delicate grey zephyr of a new shade of silvery softness which would harmonise admirably with white cap and apron. There are several varieties of navy blue twill with fancy stripes in white and red which would make up both prettily and effectively, and many others too numerous to mention.

### ARTISTIC POST CARDS.

Messrs. Beechings (Limited), of 174, Strand, are issuing a series of artistic post cards which we recommend to the notice of all correspondents. Besides being very handy and of a "save time" and money "order," these post cards are quite original and unique. They are illustrated on one half of the side with clever little sketches of London life from the brush of Miss Jessie Caudwell. The scenes, each of a different kind, are printed in colour from half tone surface blocks, and are very well reproduced by Messrs. Beechings.

### Holidays and Health.

[Readers of THE HOSPITAL in need of information about health resorts at home or abroad, or desirous of aid in forming holiday plans, are invited to send queries to EDITOR, 428, Strand, W.C. (marked "Travel" on outside of envelope), which will be answered under this section.]

### LONDON'S PLAYGROUND.

SWITZERLAND has been called the playground of Europe, and certainly the Thames fills much the same *rôle* for the Londoner. From the Greenwich excursionist to the solitary canoeist, paddling up beyond Cricklade almost to the source, many thousands daily succeed in attaining in one way or another on the river their chosen form of recreation; and, spite of the crowds which haunt the waters from early dawn to midnight, the Thames remains always, by some rare felicity peculiar only to such waterways, unspoiled by either fashion or vulgarity. The lover of peace and seclusion may be jarred momentarily by a discordant irruption of sound, but soon at the next bend the intruders are swallowed up, the swell of the passing steamer laps itself quietly to rest, the voices die away in the distance, and a stillness more perfect, because more gratefully felt, settles down between the wooded banks.

How best to enjoy it?

It is necessary, in the first place, to warn all who turn their thoughts riverwards that economy is a matter hard to compass. Seeing a glimpse from the train of charming villages dotted here and there along the stream, the unwary one innocently concludes that nothing could be a simpler or more inexpensive plan than to pass a week or two at one of these rural-looking village inns, or in one of the picturesque little labourers' cottages which line the straggling street. He is very speedily undeceived when his inquiries on the spot begin. The tiniest cottages, with a yearly rental of some £6 or £8, let during the season weekly at from 15s. a room, and in the better-sized ones £1 a room and even more is asked and obtained without difficulty. As for those fascinating river inns, their bills will not compare very favourably with those of the best hotels in London. This, it must be remembered, is at points where the railway touches the river. The wiser course is to make for villages, such as Purley, Basildon, Moulsford, Crowmarsh, or Culham, just off the line of rail, where accommodation may be found at far cheaper rates. The boating is not, perhaps, made quite so easy, but it is always practicable. A word of caution may not be amiss about hiring boats. At the up-river boat-yards (excepting Oxford, where they are cheap enough) the prices are nearly double those asked lower down, and as much as half-a-guinea a day is an ordinary demand for a good skiff.

Those who have only a limited time to spare and want to be on the river every available moment cannot do better than take the train to Oxford and row leisurely down. The distance is just a hundred miles to Mortlake, and as eighteen miles a day is a fair average down stream it may be done very comfortably by spending no more than five nights at villages on the way.

But there are many who love the river without necessarily wanting to be on it; the up-river reaches are admirably suited to the purposes of the walker, and, indeed, their peculiar beauties are never entirely laid open to those who survey them only from the surface of the water. An excellent walking route is attained by starting from Windsor and following the course of the river up to Lechlade. The river distance is again a hundred miles, but there are numerous short cuts which lure the wayfarer from the beaten track, and many miles may be saved where the river makes wide curves, as, for instance, on leaving Henley, where a nine miles' cut through fragrant pine woods brings the pedestrian out at Goring, a distance of twenty miles higher up the river. As a rule, however, it will be found far more agreeable to follow the river in all its windings, ferried from side to side as the path changes, turning aside occasionally to see some interesting bit of architecture with which the region abounds, and choosing always for the night's halt some village remote from railway station and fashion. Progressing in this manner, freed from all anxiety about wind or stream, and unshackled by tow-line or oar, the walker falls into the belief that this, after all, is the acme of enjoyment on the Thames, and that sailing vessel, punt, canoe, and skiff exist solely to heighten the exquisite and ever-changing effects encountered at every step.

### French Schools for Trained Nurses: Their Origin and Organisation.

By MADAME W. VIGNAL.

### IV.

THE Salpetrière School was founded in 1878, and is both a primary school (*école primaire*) and a professional school. The Salpetrière primary school was under the superintendence of Mdlle. Nicole from 1878 to 1891, and her duties were performed in the most admirable manner. Her spirit of self-sacrifice and remarkable intelligence are well known to all interested in medical philanthropy. Madame Pigeon has filled the same post since 1891, and exercises an admirable influence over the young nurses, whom she induces to study so that they may pass the examinations for the essential *certificat d'etudes*. The number of pupils who attend the lectures necessary for the above-mentioned examination varies considerably, as the following statistics will show: In 1891 there were 120 pupils; in 1892, 108 pupils; in 1893, 137 pupils; in 1894, 105 pupils. The pupils in the primary schools are divided into two sections, the amount of their acquirements determining their position. These sections are again divided into classes. The primary school is an annex of the professional school. It is very useful, and was even more so at the time when the Salpetrière schools were formed. At that period both male and female nurses were thoroughly ignorant. Many were Brittany peasants, speaking only their own *patois*, incapable of speaking, or even understanding, the French language. At the present time, although the law in relation to education is not strictly carried out, it has, nevertheless, a very marked effect, and ignorance amongst the lower classes is not so common as it was formerly. Most of the nurses who each year enter the Salpetrière School are able to read and write, and even amongst the Brittany women there are some who can show their *certificat d'étude*. Fifteen years ago the majority of the pupils at the Salpetrière School were natives of Brittany, and thoroughly ignorant; now the aspirants for the nursing profession come from all parts of France, a large proportion of them being derived from the Parisian population, which is generally less ignorant than that of the country places.

The lectures at the Salpetrière Nursing School are attended by pupils of different classes, of which the male and female nurses in the Paris hospitals constitute the most important contingent. They devote their spare time in the evening to study and to attendance at the lectures, so as to obtain the diploma. Another class of pupils, termed *externes* (outdoor pupils), is composed of midwives and of women desirous of qualifying themselves to be private nurses; also of a few ladies of good position, but these are quite the exception. Women of the upper classes who wish to qualify themselves as nurses attend the lectures given by the *Union des Femmes de France* and the *Association des Dames Françaises*, which are private philanthropic institutions, organised for helping the wounded in time of war or in stress of public calamity.

(*To be continued.*)

### Flat Foot Occurring in Nurses.

By Wm. Horrocks, M.B., F.R.C.S., Hon. Surgeon to Bradford Infirmary.

I.

Everyone, who has had experience of hospitals must have been struck with the frequent occurrence of flat foot in probationers during the early period of their training. The pain and difficulty of their progression is evident, and eventually the trouble becomes so great that the probationer is compelled to appeal to the lady superintendent, who, with the medical referee, gives, in some cases, a reluctant relief to the probationer by cancelling her engagement. One often feels much sympathy with the probationer, but the fact that the trouble has come on so soon, and will probably recur just when she is most wanted, causes a difficulty in recommending a continuance of the work. Hence it seems advisable to explain some of the causes which lead to this trouble and to indicate how it may in many cases be avoided.

Nurses generally begin their probation with considerable enthusiasm; the idea of doing really useful work, which has been long latent, now becomes a reality. The difficulty in obtaining admission into hospitals is an additional incentive and stimulant, so that the probationer begins her difficult work actuated by the best motives and zeal.

A day's work in the wards is frequently a rather severe shock, for it entails long hours of work under the eye of a ward nurse, who is probably not over careful of the newly-imported treasure, considering necessity of extracting help from her, rather than thinking of her as a stranger set to unusual work.

Added to this there is excitement in dealing with persons suffering from serious diseases and disappointment at finding the real work of a nurse so different from the ideal.

The prolonged hard work tries the probationer so much that she is glad to stay in her room during her time off duty, without attempting to get fresh air or sunlight. This necessarily affects the general health, and depression, caused by constantly breathing vitiated air, begins to show itself. Another cause of trouble is the food. When first hospital is substituted for ordinary home diet, an additional strain is thrown on the probationer. Food for a large number of people must of necessity be served somewhat roughly, and unless very closely supervised must be wanting in variety and suitability for individual tastes. The old custom of serving some of the nurses' meals in the ward kitchens is now becoming obsolete, but the vicious custom of drinking strong tea at all times is unfortunately very common. The brown teapot standing well smoked on the hob is known to all who frequent nurses' sitting rooms. The objections to this indiscriminate tea drinking are, that strong tea taken without food is injurious to the stomach—that it satisfies the appetite and takes the place of really nourishing food.

In most well-regulated hospitals the lady superintendent is present at the chief meals, and this ensures the proper cooking and serving of the food. It prevents the hurried scramble through the meal, and allows the lady superintendent to note any want of appetite amongst her charges, and she can then take steps to remedy the trouble.

The more immediate causes of flat foot are defective shoes, polished floors, and long standing. Those who look through the advertisements of nursing journals must have been struck by the singular want of sense shown in the shoes advertised for nurses. They often indicate an attempt to cramp the foot into the smallest possible space as the ultimate aim of the shoemaker. When one considers that the nurse stands for longer hours than the shop girl or domestic servant, and walks constantly on polished floors, the absurdity of wearing such shoes is evident. The high heel throws the weight of the body, not on its proper basis of support, the roots of the toes, but on the middle of the arch of the foot. Although by bending the foot it makes it appear shorter, this deception is gained by a great strain on a ligament. The shoe is very imperfectly secured to the foot, so the foot has no fixed connexion with it. Added to this the narrow sole and pointed toe cramp the front of the foot.

### For Reading to the Sick.

### SUNLIGHT.

**Motto.**

He suffered in the darkness
That we might see the sun.

**Verses.**

Jesus my heart loves dearly,
All through the darkest night,
As when the sun shines clearly,
Making my pathway bright.
—*Hymns of C. and F.*

The whole world was lost in the darkness of sin,
The Light of the World is Jesus.
Like sunshine at noonday His glory shone in,
The Light of the World is Jesus!

No darkness have we who in Jesus abide,
The Light of the World is Jesus.
We walk in the light when we follow our guide,
The Light of the World is Jesus!

Ye dwellers in darkness, with sin-blinded eyes,
The Light of the World is Jesus.
Go, wash at His bidding, and light will arise,
The Light of the World is Jesus!

No need of the sunlight in heaven, we're told,
The Light of the World is Jesus.
The Lamb is the Light in the City of Gold,
The Light of the World is Jesus!

Come to the Light, it is shining for thee;
Sweetly the Light has dawned upon me,
Once I was blind but now I can see,
The Light of the World is Jesus!
—*Songs and Solos.*

**Reading.**

The Lord is my Light and my Salvation.—*Ps.* xxvii. 1.

A Light that shineth in a dark place.—2 *St. Peter* i. 16.

We have noticed in November days that some people seemed to choose the dark dingy side of the street, while others were walking in the beautiful sunshine. Perhaps they had but a little way to go—they thought it did not matter much; perhaps they did not observe the sunny side. A little bit of sunshine makes such a difference, especially when the summer heat is over and the chilling frosts of winter come creeping on. All the more need have we to

Gather up the sunbeams
Lying all around our path.

In our lives we shall find that to most things there is a "sunny side" if we take pains to look for it. Many people, Christians even, have a wonderful knack of looking at the dark side of things. They seldom have anything bright to tell you, and there is very little brightness in their faces. They do not look as if Jesus had "made them glad." . . . . But some of God's believing children are in deep sorrow, and where is the sunny side for them? . . . . These are they who one day will "comfort others with the comfort wherewith they themselves have been comforted of God." Noah had only one window and it was "above"! It is well to keep the mists off this window! When all was death and desolation around, "God remembered Noah," and was not that enough? . . . .

Even God's children in this earthly life have shadows cast upon their lot, though the hills beyond are flooded with the glorious sunshine! A good deal of shadow perhaps, but a shadow cannot harm! "Nobody is afraid of a shadow, for a shadow cannot stop a man's pathway even for a moment. The shadow of a dog cannot bite, the shadow of a sword cannot kill."—*Spurgeon.* "Yea, though I walk through the valley of the shadow, I will fear no evil, for Thou art with me." And the sunny land is not far away! No shadows yonder, all light and song. "The city had no need of the sun, neither of the moon, to shine in it, for the glory of God did lighten it, and the Lamb is the light thereof."

B. Campell.

### Everybody's Opinion.

[Correspondence on all subjects is invited, but we cannot in any way be responsible for the opinions expressed by our correspondents. No communications can be entertained if the name and address of the correspondent is not given, or unless one side of the paper only be written on.]

### NURSING ASSOCIATIONS.

MISS AMY HUGHES writes: I regret that Lord Kinnaird has construed a simple relation of facts into an underhand attack upon the society in which he is so much interested. I made no statement of any kind regarding the work of this nursing association, nor, to my knowledge, any "mis-statement." I merely corrected the erroneous impression liable to be produced by the newspaper report (from which I quoted in my letter to THE HOSPITAL of 13th inst.) that this association was in any way connected with the Metropolitan Association. The Central Home is now the Central Training Home of the Queen's Nurses, and as the work of the Queen's Institute varies materially from that of many other societies, I gave a short sketch of its principal features. It is always a matter of regret when the personal element is introduced into a general discussion, but I would like to state I have had some years' practical experience of the work of these nurses before the conference to which Lord Kinnaird alludes. Also that an inquiry at headquarters would show that the work of the Central Home was neither "volunteered" to St. Bartholomew's Hospital, nor is it now being performed voluntarily.

### HOLIDAYS.

"ONE WHO IS LOOKING FORWARD TO A HOLIDAY," writes: In a paragraph in the *Poor Law Officers' Journal* I have read with interest as follows: "Miss Richardson, a nurse in the service of the Ballymoney Guardians, has a prospect of getting a retiring allowance of £15 per annum. The Guardians will shortly consider the recommendation of a committee which is to this effect, and we hope they will endorse it. When it is considered that Miss Richardson has given 25 years' service, during which time she had only one holiday, few will be disposed to deny that her case is a deserving one." Curiosity is indeed raised as to the motive which produced in Miss Richardson either contempt for holidays or such singular powers of devotion to duty. The nurses of the institution to which I belong begin as soon as Christmas festivities are over to look forward to their annual leave, and great is the excitement as the time draws near—every week, nay, every hour, is counted. Under the Poor Law salaries are not enormous, but had this lady known of the magnificent annuity in store for her she would surely have taken more than one solitary holiday in a quarter of a century! All thoughts of providing for old age would have vanished, and holidays could have been enjoyed without dread of the future. This is the only solution I, to whom my annual leave is an absolute necessity, can give. As the annuity it still in prospect perhaps the Board of Guardians may vote that an addition of £10 may be made to the sum already proposed.

### NIGHT NURSING.

MISS MEYRICK writes: At a recent meeting of the Aylesbury Board of Guardians they decided that a night nurse for the Poor Law Infirmary was unnecessary, notwithstanding the recommendation of the Local Government Board, who, in their wise circular, held a different opinion. The resolution proposing the appointment of a night nurse was rejected, and an amendment carried to the effect that a night nurse should be employed when necessary. Surely a nurse at night is as necessary as a nurse in the day! Patients who need nursing by day very often need it much more by night, and it is sad to think of wards full of sick people, many of them helpless, and some of them imbecile, left without care at night. It is a pity that these gentlemen cannot be roused to a due sense of their responsibility to their poorer brethren, and induced to give them what is really a necessity. The aged and sick poor have a claim upon us which the idlers and vagrants have not. If they are afraid of increasing the rates, even in that view of the matter their course of action is penny wise and pound foolish, for extraneous nursing help, got in as often as a need is recognised, would cost more in the end and not be nearly so efficient as a permanent nurse, who would assist in washing helpless patients in the morning. The Master's opinion of the efficiency of the day nurse was taken, instead of consulting the medical officers on the matter. Now, however good a master of a workhouse may be, what possible value can his opinion be on a question of nursing? It is disappointing after all that has been done on nursing questions to find such antiquated ideas still existing in a county so near to London.

### Care of the Sick in Alexandria and Cairo.

### V.—THE GERMAN HOSPITAL.

THIS hospital is managed by Deaconesses, which prepared us for the cleanliness and order of its long cool corridors and its cheerful wards. It is surrounded by a large garden with all the luxurious vegetation of the East, and the demure, kind-hearted little women in close white caps who flitted about with calm quiet faces, gave a restful feeling. The hospital is open to Arabians, Greeks, in fact to all nations, for ordinary diseases and accidents, but for infectious cases it is used only by English and Americans; but surgical cases preponderate. There are twelve Sisters, five servants and six helps, and at the time of our visit there were sixty patients, the full number being ninety.

The average percentage of deaths per annum is 7.

The men are on the upper floor and the women on the lower, and all have to submit to a bath on entering whether they like it or not; the Arabs of course object to this immersion.

All the wards were supplied with books and pictures; the private rooms having two beds and the third class six. The black and white tiled floors looked cool and clean, and the glimpses of the patients' garden with its poinsettias and palms and vivid blue sky through the open windows gave a cheerful air to the whole. We crossed a terrace covered with a superb purple creeper which transformed it into a fairy bower.

The kitchen and scullery left nothing to be desired, and the laundry was well supplied with all necessary machinery, while the numerous bathrooms showed that cleanliness was considered a virtue. There were little pantries or sculleries to each floor where beef tea, hot water, or poultices are procured. Children are placed with the adults; and in one of the wards we saw an English sailor who said he was comfortable and kindly cared for.

There is a well-stocked linen-room, and a sitting-room for the Sisters where they go from eight to nine. One sister watches all night. Sister Barbara, the superintendent, has been there a quarter of a century, and what she did at the time of the bombardment is a matter of history. The hospital has grants from three Governments, as follows: From the Egyptian, £60; from the Prussian, £50; and from the English, £30.

The Sisters have plenty to do, but they take things quietly and get through their work systematically and with kind sympathy for the sick.

### The Book World for Women and Nurses.

An Australian in China. By G. E. Morrison. (London: Horace Cox. 1895.)

Mr. Morrison's book is one of a really interesting nature, and repays a careful reading. It is, of course, as its title says, a book of travel, but it is something more than an ordinary geographical primer—there is a great deal of thought and reflection on the writer's part, and the subjects he deals with are out of the common. The book teems with incidents. Perhaps the two main features which strike the reader are those dealing with the missionary and the medical work in China. These, Mr. Morrison puts plainly before us. With reference to the latter, scattered throughout the pages, we find many entertaining observations amongst other things. At the Mission Hospital at Chungking—which the author describes as a well-equipped Anglo-Chinese building attached to the city wall—there is a separate compartment walled off for the treatment of opium smokers who desire by forced restraint to break off the habit. Three opium smokers were in durance, he tells us, at the time of his visit there, suffering, on their own confession, from the opium habit. These men, having freely expressed the desire of their hearts to be cured, were received with welcome and placed in confinement. Alas for the futility of human aid in all such cases! Every effort was made to wean them from the habit which they alleged had seized them "in a death grip." Attentive to the teacher and obedient to the doctor, the poor victims gave every hope of being early admitted into church fellowship. But one night the desire to return to the drug became irresistible, and strangely, remarks Mr. Morrison, "the desire attacked all three men at the same time and on the same night, and they escaped together. Sadly enough, there was in this case marked evidence of the demoralising influence of opium, for when they escaped they took with them everything portable they could lay their hands on." Excellent medical work is done in this hospital, and from the first annual report, just published by the surgeon in charge, an M.D. from the United States, the two following quaint items are given us:—

Medical Work: "Mr. Tsang Taotai was an eye-witness to several operations, as well as being operated upon for internal piles."

Evangelistic Work: Mrs. Wei, in the hospital for suppurating glands of the neck, became greatly interested in the truth whilst there, left a believer, and attends Sunday service "regular," walking from a distant part of the city each Sunday. We regard her as very hopeful, and she is reported by the Chinese as being very "warm-hearted." She will be converted when the first vacancy occurs in the nursing staff. No pharmacopæia is more comprehensive, the writer tells us, than is the Chinese; the amount and variety of drugs collected together in Chaotong City is extraordinary. As to the doctors themselves, he says: "No English physician can surpass the Chinese in the easy confidence with which he will diagnose symptoms that he does not understand." A curiously dominating fatalism characterises the medical practitioner in the Celestial Empire. "There is medicine for sickness, but none for fate" is one of their proverbs, and another is 'Medicine cures the man who is not fated to die!" The professional knowledge of a Chinese doctor largely consists in his ability to feel the pulse, or, rather, the innumerable pulses, of a Chinese patient. These pulses, according to our author, vary in a manner no English doctor can conceive of. He quotes, in a really humorous manner on page 107, the seven kinds of pulse which presage approaching death, as an example of which we give the following, which will sufficiently serve to show the physician's mastery of the theory of this art. "If the motion of the pulse resembles the pace of a frog when he is embarrassed in the weeds, death is certain."

For ague, which, as we all know, is one of the common ailments of the country, there is an admirable prophylactic at hand against it: "Write the names of the eight demons of ague on paper, and then eat the paper with a cake; or take out the eyes of the paper door-god and devour them; this remedy never fails." In China all the doors nearly have gods above them.

Mr. Morrison, being a medical man, is naturally interested in medical work on his travels through China, and his observations on what he sees are to the point. Then, as to the other noticeable feature in his book—the missionary work as he sees it—there is a great deal said on this subject too; but there is little enough unfortunately of an encouraging nature to the workers. "I believe," says Mr. Morrison, "that it is now universally recognised that the most difficult of missionary fields, incomparably the most difficult, is China," and as a sound reason for this state of things, the author reminds us of "the prepossessions and cherished judgments" of the Chinese, "which are the growth of millenniums."

The chief of all virtues in China is filial piety; the strongest emotion that can move the heart of the Chinaman is the supreme desire to follow in the footsteps of his father. Conversion with him means not only eternal separation from the father who gave him life, but the "immediate liberation of his ancestors from a life of beggary, to inflict sickness and all manner of evil on the neighbourhood."

With such a people the missionaries have a hard enough problem to face. In his book, Mr. Morrison puts this before us in a trenchant manner. But there is so much brought before our eyes in this work it is not easy to single out any separate parts as examples. We have dealt with two points only—two among the many hundreds the author discusses. And geographically, too, the book is of much interest; we follow the writer's travels with a sense of almost personal realization, furthered as this is by the presence of numerous illustrations and a rough sketch map of China and Burmah, showing us the author's route from Shanghai to Rangoon.

The Honourable Mrs. Spoor. By Arabella Kenealy. (London: Digby Long. 1895.)

"My dear, who was this Mrs. Spoor before Spoor married her?" is the question asked by all friends concerning the heroine of the novel, who is one of Society's brilliant successes. To the reader Miss Kenealy vouchsafes a more definite enlightenment as to the previous circumstance of her principal character, the less said of which circumstances perhaps the better. The book gives us the history in a wholly unreserved manner of the moral and mental break-up of a woman who has had a past. It is not a pleasant past, and neither is it pleasantly described; but there is a certain originality in the plot which redeems it from commonplaceness. The Honourable Mrs. Spoor is introduced to us first at what may be called the zenith of her fame, her social fame. Everything was going well; she is admired, even respected, the wife of a great county magnate. Her past is forgotten—or rather unknown. All that is asked is asked in a natural feminine curiosity, "Who was this Mrs. Spoor?" But from her pinnacle on the social ladder the heroine is shown to us in a gradual descent—tottering backwards, her power gone to conquer the assailings of her former self. All this descent, if one may so speak, is due to the fact that the regenerated woman is brought face to face with a pair of maidenly eyes. "Such eyes!" they are described as being. The incident is a trivial one in itself, and too much importance is attached to it. But be that as it may, the gaze of an innocent girl had a dominating effect upon the woman, and out of it is evolved a clever, if too realistic a story. For the book is nothing if not realistic, to use the word in its narrowest and generally accepted sense Miss Kenealy has treated her subject from a physiological standpoint, through which medium she appears to look on life, and this is obtruded a little too much upon her readers.

## Figures and Tables

**Fig. 39 f1:**
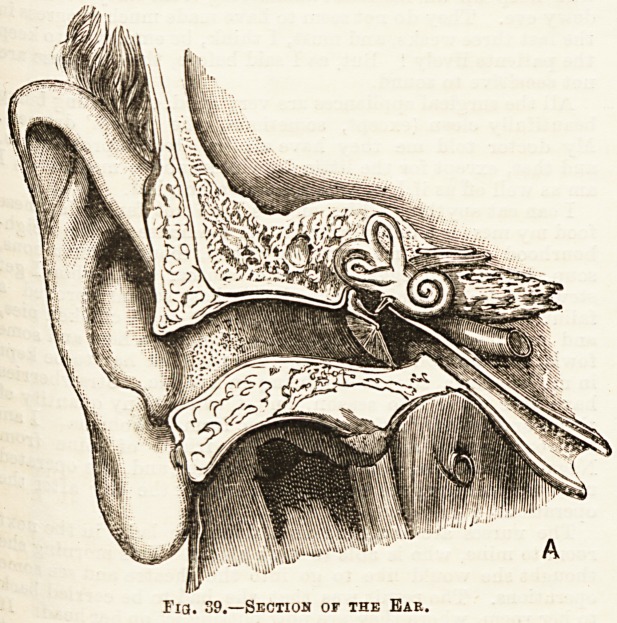


**Fig. 40 f2:**
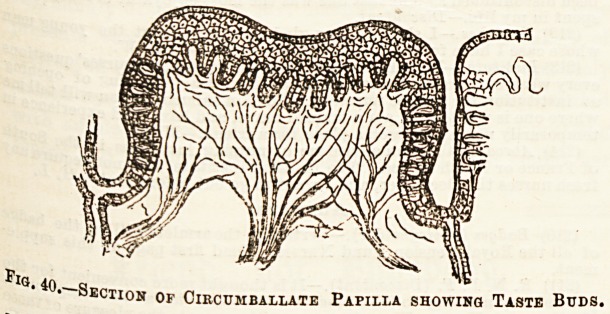


**Fig. 41 f3:**
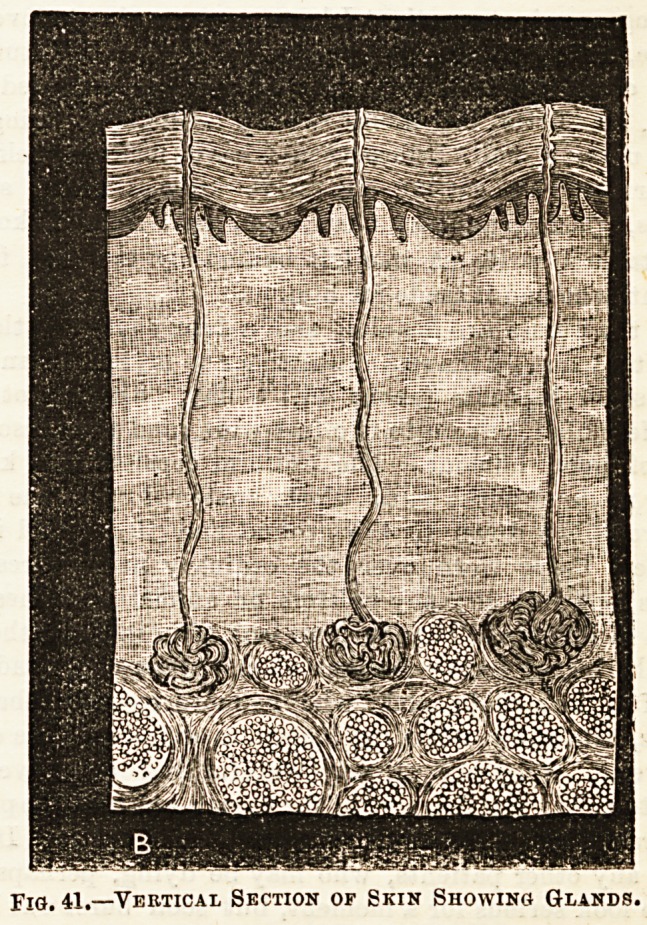


**Figure f4:**
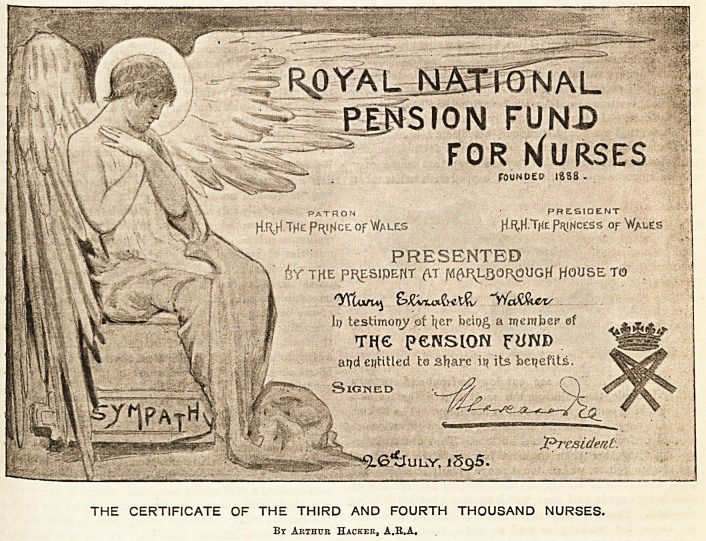


**Figure f5:**